# Tristetraprolin expression and microRNA-mediated regulation during simian immunodeficiency virus infection of the central nervous system

**DOI:** 10.1186/1756-6606-6-40

**Published:** 2013-09-02

**Authors:** Jonathan Liu, Jeanne M Sisk, Lucio Gama, Janice E Clements, Kenneth W Witwer

**Affiliations:** 1 Department of Molecular and Comparative Pathobiology, The Johns Hopkins University School of Medicine, 733 N. Broadway, Miller Research Building Rm. 829, Baltimore, MD 21205, USA; 2 Department of Neurology, The Johns Hopkins University School of Medicine, 733 N. Broadway, Miller Research Building Rm. 829, Baltimore, MD 21205, USA; 3 Department of Pathology, The Johns Hopkins University School of Medicine, 733 N. Broadway, Miller Research Building Rm. 829, Baltimore, MD 21205, USA

**Keywords:** Cytokine, RNA-binding protein, Tristetraprolin, microRNA, Human immunodeficiency virus, HIV-associated neurocognitive disorder

## Abstract

**Background:**

The RNA-binding protein tristetraprolin (TTP) participates in normal post-transcriptional control of cytokine and chemokine gene expression, dysregulation of which contributes to the HIV-associated neurocognitive disorders. Transcriptional and post-transcriptional regulation of TTP has been described, including regulation by microRNA-29a. In the simian immunodeficiency virus (SIV) model of HIV CNS disease, control of cytokine/chemokine expression coincides with the end of acute phase infection. This control is lost during progression to disease. In this study, we assessed TTP regulation and association with cytokine regulation in the brain during SIV infection.

**Results:**

Quantitation of TTP expression over the course of SIV infection revealed downregulation of TTP during acute infection, maintenance of relatively low levels during asymptomatic phase, and increased expression only during late-stage CNS disease, particularly in association with severe disease. The ability of miR-29a to regulate TTP was confirmed, and evidence for additional miRNA targeters of TTP was found. However, increased miR-29a expression in brain was not found to be significantly negatively correlated with TTP. Similarly, increased TTP during late-stage disease was not associated with lower cytokine expression.

**Conclusions:**

TTP expression is regulated during SIV infection of the CNS. The lack of significant negative correlation of miR-29a and TTP expression levels suggests that while miR-29a may contribute to TTP regulation, additional factors are involved. Reduced TTP expression during acute infection is consistent with increased cytokine production during this phase of infection, but the increases in TTP observed during late-stage infection were insufficient to halt runaway cytokine levels. While antisense inhibitors of the post-transcriptional targeters of TTP identified here could conceivably be used further to augment TTP regulation of cytokines, it is possible that high levels of TTP are undesirable. Additional research is needed to characterize members of the miRNA/TTP/cytokine regulatory network and identify nodes that may be best targeted therapeutically to ameliorate the effects of chronic inflammation in retrovirus-associated CNS disease.

## Background

The advent of effective antiretroviral therapy for HIV-1 in the mid-1990s dramatically improved life expectancy and quality [1]. However, as individuals live longer with HIV, the HIV-associated neurocognitive disorders (HAND) remain a growing problem [2,3]. HAND severity ranges from asymptomatic neurocognitive impairment to minor motor/cognitive disorder to HIV-associated dementia [4], and its effects span several neurocognitive domains, including attention/working memory, motor skills, and executive functions [5,6].

The growing prevalence of HAND dictates a need to understand the underlying disease mechanism, which have not been fully elucidated but are likely multifactorial. Neurotoxicity of viral products [7,8] and prolonged exposure to inflammatory cytokines/chemokines are probable causes [9-11]. Innate immune responses during acute phase infection may also cause damage or influence predisposition to disease [12,13]. Because longitudinal monitoring of the central nervous system (CNS) is difficult or impossible in human cohorts, our group developed a simian immunodeficiency virus (SIV) macaque model of HIV CNS pathogenesis [14,15]. In this model, pigtailed macaques (*Macaca nemestrina)* are dual inoculated with SIV/17E-Fr, a neurovirulent clone, and SIV/ΔB670, an immunosuppressive swarm. An initial acute phase, like that seen in HIV, lasts for the first 10-14 days post infection (dpi), with many measures of innate immune response peaking around 7 dpi [12]. This is followed by a latent or “asymptomatic” phase. Pronounced CD4+ T cell depletion and motor deficits appear by approximately 35 days dpi and are monitored until 84 dpi, by which time more than 90% of infected macaques have developed CNS disease and AIDS [15]. Patterns of cytokine expression during SIV infection suggest that, at terminal stages of disease, high and sustained cytokine levels contribute to neurodegenerative pathologies. Transient acute phase cytokine expression may itself cause lasting damage or set the stage for neurodegeneration. Accordingly, brain cytokine regulation is of central importance in CNS disease progression.

Tristetraprolin (TTP, also known as zinc finger protein 36 homolog, ZFP36) has been characterized as an anti-inflammatory and anticarcinogenic protein that is also involved in differentiation processes [16,17]. TTP is thought to act primarily through post-transcriptional regulation of messenger RNA [18,19]. TTP binds to and destabilizes transcripts with 3′ untranslated regions (3′ UTRs) that contain AU-rich elements (AREs) [20-22], including those of CCL2, IL-6, IL-10, TNF-α, iNOS, and many other inflammatory mediators. Post-transcriptionally, TTP activity is regulated by post-translational phosphorylation, and a cellular microRNA (miRNA) has been reported to modulate TTP abundance [23,24]. miRNAs are short, single-stranded RNA molecules—often 22-23 nucleotides in length—that regulate host and viral gene expression [25-28]. Incorporated into the cytosolic RNA-induced silencing complex, miRNAs bind transcripts through partially complementary sequences known as miRNA recognition elements (MRE), usually located in the 3′ UTR of the target transcript [25,26]. Subsequent regulation can be achieved by transcript cleavage and degradation, inhibition of translation, and cellular sequestration of transcripts.

Because TTP has been reported to regulate many cytokines that we previously observed to be coordinately regulated in SIV infection [12], and since miRNAs have various associations with CNS disease [13,29-32], we investigated the expression of TTP and potentially related miRNAs in our HIV CNS disease model. We then queried the role of TTP in cytokine regulation during SIV disease, as well as the influence of miRNA expression on TTP levels. We report here that TTP is differentially regulated during the course of SIV infection and that acute phase downregulation of TTP is mechanistically consistent with increased cytokine levels recorded during this phase. However, during the general dysregulation of late-stage disease, increased expression of TTP is apparently insufficient to halt damaging levels of cytokine production. We also confirm that miR-29a can indeed regulate the TTP 3′ UTR, but that consideration of other factors is needed to explain TTP modulation in the CNS. Finally, we present evidence for several additional miRNA members of a complex interaction network governing TTP, miRNA, and cytokine expression.

## Results

### TTP mRNA is differentially expressed during viral pathogenesis

**Figure 1:**
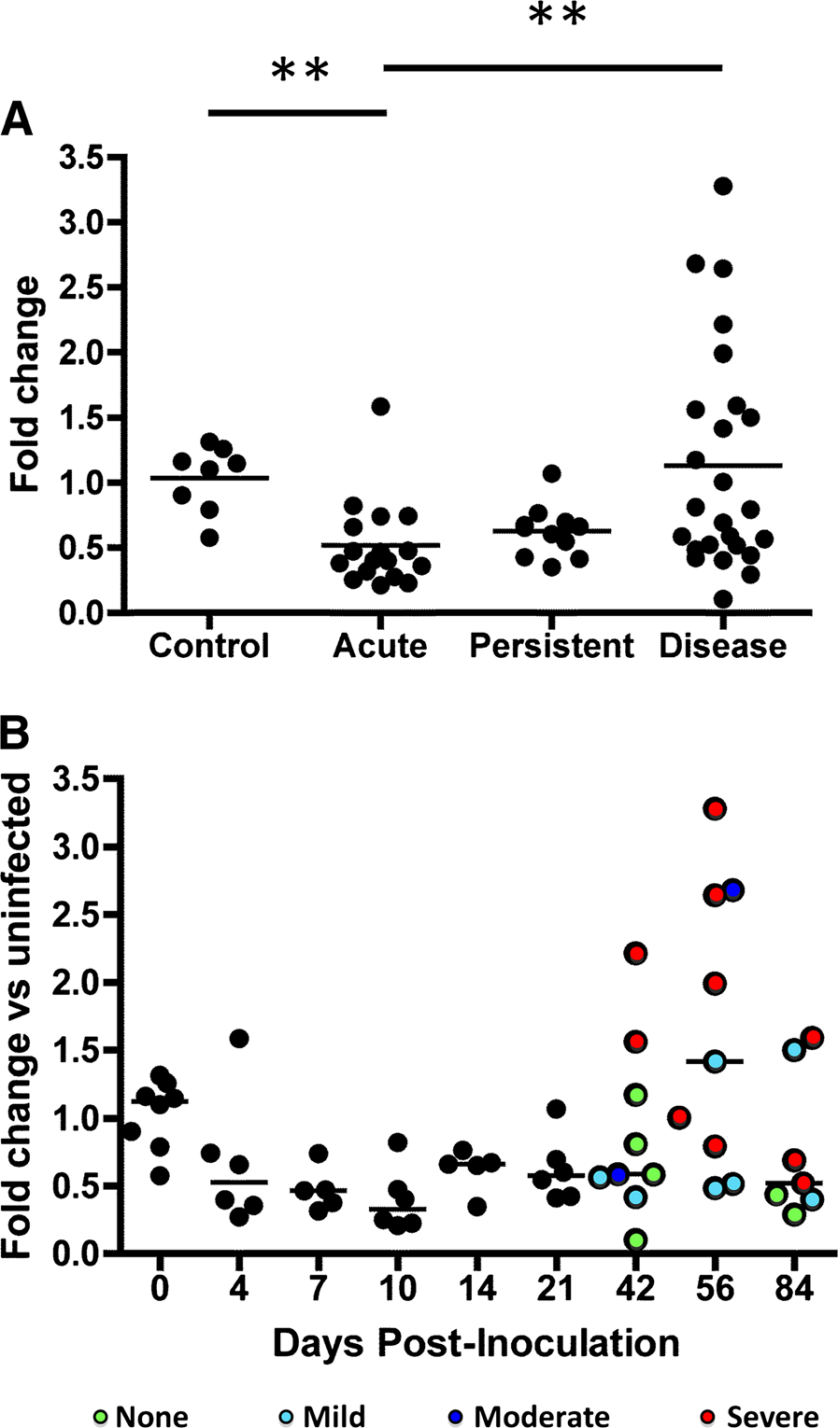
**Tristetraprolin mRNA expression during SIV infection.** TTP was quantitated by TaqMan assay using total RNA isolated from thalamus of control, uninfected (‘0’) or SIV-infected macaques at different stages of infection and disease **(A)** and at the indicated number of days post-inoculation **(B)**. Data were normalized by delta-deltaCt method, with 18S rRNA as the control, and graphed relative to the average expression of the control group. Bars represent mean **(A)** or median **(B)** fold change in each stage or at each time point. Shown for late-stage infection **(B)**, CNS disease severity as determined by pathology examination is denoted by color: green = none, turquoise = mild, blue = moderate, red = severe. Inset: data sorted by stage of infection. Acute phase comprises days 4-10; persistent includes time points with lower viral load and cytokine expression (14 and 21); late-stage disease begins at day 42. ** denotes p < 0.01 by Kruskal-Wallis test with Dunn’s multiple comparison test.

TTP mRNA in SIV-infected brain (thalamus) was quantified during several stages of SIV infection in macaques (Figure 1A), comparing control, uninfected animals with acute (4, 7, 10 dpi), persistent/asymptomatic (14, 21 dpi), and late-stage infection (42, 56, 84 dpi). Thalamus was chosen because SIV replication occurs at high levels in thalamus and SIV lesions are observed most frequently in thalamus, basal ganglia, and parietal cortex [14]. The TTP transcript is downregulated relative to uninfected controls during acute infection (p < 0.01, Kruskal Wallis test with Tukey’s post-test) and, in most individuals, appears to be present at a decreased level throughout infection. However, a trend towards recovery to control levels is seen at the end of acute phase, when TTP levels are no longer significantly different from control, while TTP abundance is significantly lower in acute versus late stage disease. As revealed by assessment of CNS disease severity at specific time points post-infection (Figure 1B), this difference is driven by the production of larger amounts of TTP transcript by individuals that developed severe CNS disease. Indeed, during late-stage infection, subjects that had severe or moderate CNS disease had significantly more TTP mRNA than those with no or only mild disease (p = 0.0021, Mann Whitney test).

### miR-29a targets the 3′UTR of TTP

**Figure 2:**
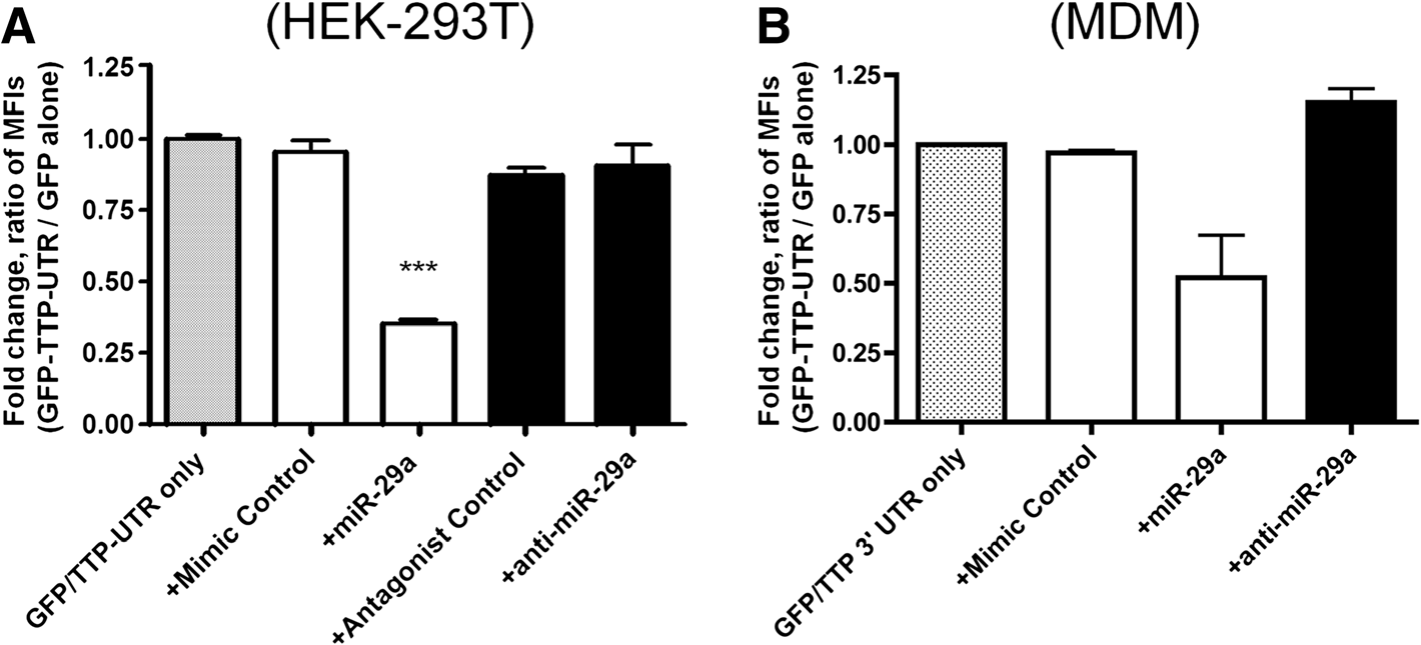
**miR-29a-mediated regulation through the TTP 3′ UTR.** HEK-293 T cells **(A)** or monocyte-derived macrophages **(B)** were transfected with reporter plasmids (GFP-TTP-3′ UTR or GFP empty vector), red fluorescent protein (RFP) transfection control, and small RNA molecules as indicated (scrambled mimic control, miR-29a mimic, antagonist control, or miR-29a antagonist). 24 hours post-transfection, fluorescence of cells transfected with reporter and empty vector were determined by flow cytometry. **A)** Exogenous miR-29a caused a significant reduction (***, p < 0.0001 by *t*-test, p < 0.01 by ANOVA) in the normalized fluorescence ratio of HEK-293 T cells transfected with the GFP-TTP-3′ UTR construct vs. GFP vector alone. **B)** Because of sample-to-sample variability, the consistent decrease in normalized GFP-TTP 3′UTR fluorescence in the presence of exogenous miR-29a in macrophages approached but did not reach statistical significance as determined by ANOVA (p > 0.05). Error bars are standard error of the mean.

Both RNA-binding proteins [33]—such as TTP—and miRNAs are known to regulate transcripts, usually through elements in the 3′ UTR. Moreover, miR-29a was previously shown to target the TTP 3′ UTR directly [23]. We therefore examined miR-29a expression in SIV-infected brain to determine whether this miRNA might play a role in regulation of TTP during lentiviral infection and CNS disease. Using in silico analyses that combined several miRNA target prediction algorithms, we verified the previously reported response of the TTP 3′ UTR to miR-29a [23]. In a fluorescent reporter system comprising HEK-293 T cells transfected with green fluorescent protein (GFP) reporter constructs with or without the TTP 3′ UTR, miR-29a-mediated regulation of TTP was confirmed (Figure 2). Flow cytometry showed a greater than two-fold downward shift in the mean intensity of cells transfected with the TTP 3′ UTR construct compared with cells containing the GFP vector alone, suggesting that HEK-293 T cells contain endogenous factors, including but not necessarily limited to miRNAs, that exert regulatory effects through the TTP 3′ UTR. Compared with the ratio of fluorescence of cells containing the GFP-TTP- 3′ UTR construct versus GFP vector alone, co-transfection with a miR-29a mimic, but not a scrambled control RNA (“mimic control”), caused a further decrease in mean fluorescence of cells transfected with the 3′ UTR construct (Figure 2A), while neither miR-29a mimic nor mimic control exerted significant effects on the GFP-only construct. Together, these results confirmed that miR-29a is among the regulatory molecules that target the TTP 3′UTR. However, co-transfection of an antisense miR-29a antagonist and the TTP UTR construct did not result in a significant increase in mean fluorescence intensity when compared with transfection of a control antagonist or no antagonist (Figure 2A). This could be due to a low endogenous level of miR-29a. Additionally or alternatively, although exogenous miR-29a clearly enhances 3′ UTR-mediated suppression, additional miRNAs or other factors are capable of enforcing suppression in a commonly used cell line, even during suppression of miR-29a.

### Transfection of microRNAs into human macrophages affects TTP levels

We next characterized the effects of miR-29a mimics and inhibitors on TTP expression in cells of the monocyte/macrophage lineage. Monocyte-derived macrophages were chosen for two reasons. First, macrophages are central actors in immune responses and viral replication and entry into to the brain in our SIV encephalitis model as well as in HIV-1 infection of brain. Second, previous work indicated the involvement of macrophage TTP in regulation of cytokines such as TNF-alpha [34-36]. Third, miR-29 species are upregulated in macrophages during differentiation [37] and in early SIV infection [38]. Flow cytometry of macrophages that were co-transfected with the GFP-TTP 3′UTR reporter vector and small RNA molecules showed that a miR-29a mimic, but not a control RNA, reduced mean fluorescence intensity by 20-70%, i.e. consistently, but with considerable inter-donor variability (Figure 2B). As with the HEK-293 T experiments, however, only a slight increase in fluorescence was seen upon transfection with a miR-29a antagonist, suggesting redundancy of 3′ UTR-binding sites in macrophages. We also performed an experiment in which mimics and inhibitors of miR-29a were transfected into primary macrophages without a reporter vector to assess the effects of the miRNA on native protein. Semi-quantitative analysis of Western immunoblots for native TTP protein revealed that transfection of miR-29a inhibited TTP production, whereas anti-miR-29a resulted in a 4-fold increase of protein (Additional file 1: Figure S1). We thus confirmed that miR-29a is a regulator of TTP, including in the disease-relevant setting of the macrophage.

### Fluctuation of thalamic miR-29a levels during progression to SIV CNS disease

**Figure 3:**
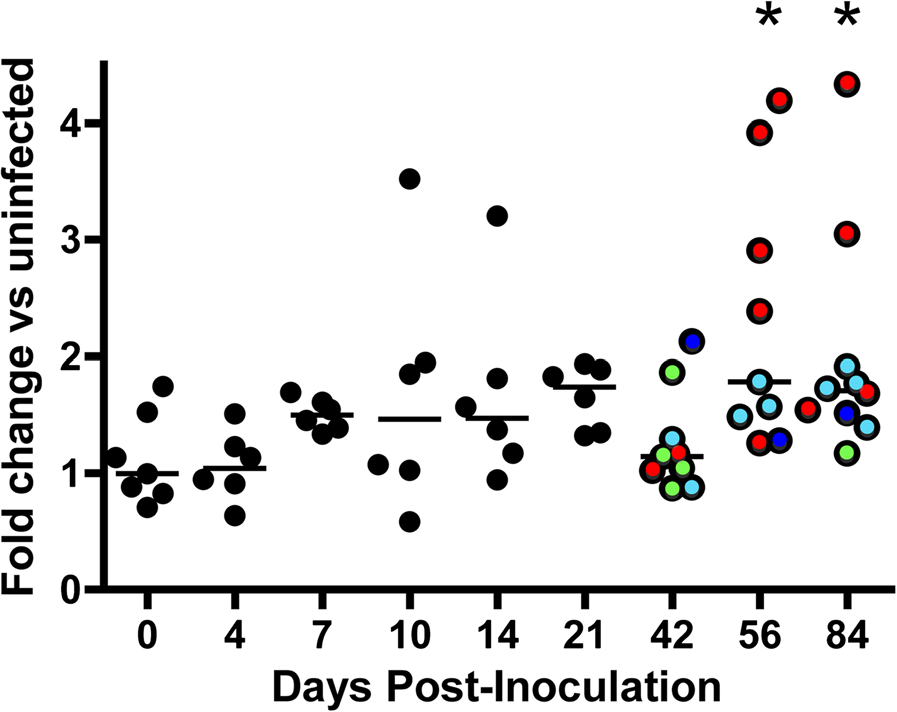
**Longitudinal expression of miR-29a in SIV-infected brain.** Expression of miR-29a was determined by miRNA stem-loop RT-qPCR using the delta-deltaCt method with normalization to snRNA U6 (similar results were obtained with normalization to the geometric mean of multiple invariant miRNAs—data not shown). Fold changes relative to the average of the control animals (‘0’) are shown. Bars represent the mean at each time point. Color coding of severity during late-stage infection is as described for Figure 1. Statistical significance was assessed for each time point versus control by Kruskal Wallis test with Dunn’s test for multiple comparisons; * represents p < 0.05.

To assess the relationship of miR-29a and TTP in brain, miR-29a levels were measured in thalamus in the SIV/macaque model, spanning the course of viral pathogenesis. The mature human and pigtailed macaque miR-29a sequences are 100% identical. Although there was an apparent trend towards increasing miR-29a values during infection, and group means were significantly different (p < 0.01, Kruskal Wallis), relatively constant mean levels of miR-29a were maintained at least through latent infection, and in almost all animals, miR-29a abundance was within two fold of the average of control, uninfected animals (Figure 3). However, when compared with controls, there were significant increases (p < 0.05 by Kruskal Wallis test) in the group means of the 56 and 84 dpi late-stage animals (as well as for all late-stage groups together). There was also substantial variation within each group. As with TTP mRNA levels, miR-29a expression tended to be highest in animals with severe disease.

### Relationship of miR-29a and TTP mRNA levels

An inverse correlation of miR-29a and TTP expression might be expected in the case of an exclusive, classic regulatory relationship of the miRNA with its target. However, no significant correlation was found in our model across time points (data not shown). Analyses focused on specific phases of infection (acute, persistent/asymptomatic, and late-stage disease) similarly found no negative correlations of miR-29a with TTP. As noted, miR-29a and TTP levels were highest in late stage disease, and a positive correlation (R = 0.58, data not shown) was in fact observed between the two analytes at 84 dpi.

### Novel miRNA regulators of TTP

We sought to identify additional miRNA candidates for TTP 3′ UTR regulation for three reasons. First, although exogenous miR-29a inhibitors allowed greater production of native TTP protein in macrophages (Additional 1: Figure S1), our reporter experiments in both 293 T and primary macrophage culture indicated that miR-29a was not the only regulator of the TTP 3′ UTR. Second, as described, we did not observe an inverse relationship of TTP and miR-29a levels in vivo, as one might expect if miR-29a were a major physiologically relevant regulator of TTP during SIV infection in brain. Third, relationships of TTP with additional miRNAs in more complicated regulatory networks have been proposed in the recent literature [39,40]. Three target prediction algorithms were used to generate a list of candidate miRNAs, which were ranked based upon the number of predicting algorithms and the number of predicted MREs in the 3′ UTR for each miRNA.

**Figure 4:**
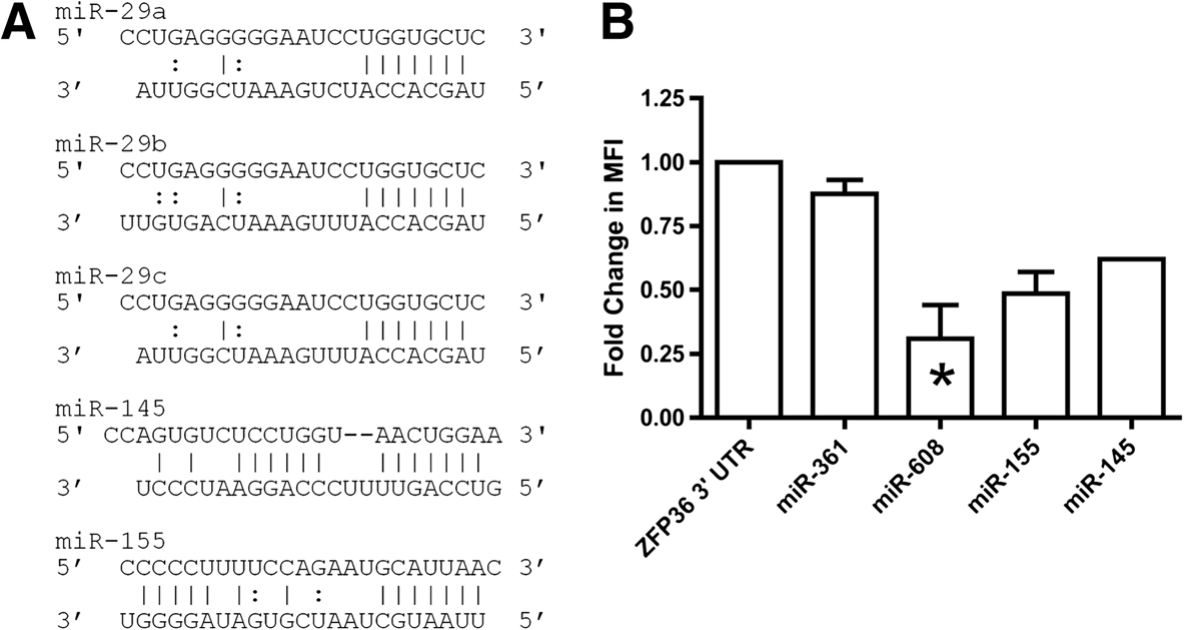
**Novel interactions of miRNAs with TTP 3′ UTR elements. A)** Alignment representing the previously-described miR-29a/TTP 3′ UTR interaction, along with alignments for additional miR-29 family members and a novel predicted interaction with miR-145. In each alignment, TTP (top) and miRNA (bottom) canonical base pairings are shown with ‘|’, while non-Watson-Crick “wobble” pairing of G and U is shown as ‘:’. **B**. Reduction in mean fluorescence intensity (MFI) of the TTP (ZFP36) 3′ UTR in the presence of the transfected synthetic miRNAs (HEK-293 T cells). Error bars are standard error of the mean.

Among miRNAs detectable in brain (unpublished data), there were approximately 50 candidates, and requiring at least one perfect seed match narrowed the field to 18. Interestingly, although miR-29a was predicted by one of the programs (TargetScan), it did not appear to be the strongest match by free energy criteria. The TTP MRE seed match for miR-29a (or, indeed, for any miR-29 family member) is highly conserved among primates, from human to marmoset. However, there is little interaction outside the seed region, with one potential G-C pairing and two (miRs-29a and -c) or three (miR-29b) G:U wobble pairs (Figure 4A). As a result, target prediction algorithms that considered interactions outside the seed region, but not site conservation, did not necessarily return miR-29 as a TTP interactor. According to TargetScan, which did predict miR-29, the context score percentile for the interaction is 55 or 59 for 29a/c and 29b, respectively. miR-145 and miR-155 target interactions had context scores of 91 and 42, respectively (Figure 4A). In contrast, miR-361-3p had four predicted recognition elements in the TTP 3′ UTR that were predicted by at least one program, including three perfect seed matches (alignments not shown). Although TargetScan context score percentiles for the human sequences were 55, 65, and 71, the target sequences for miR-361-3p were not well conserved, there was little or no interaction outside the seed region, and the seed interaction regions were low complexity G repeats. miR-608, similarly, relied on low-complexity sequence recognition.

We selected four miRNAs for transfection into macrophages along with the TTP 3′UTR reporter construct: miRs-145, -155, -361-3p, and -608. Despite numerous predicted binding sites in the TTP 3′ UTR, miR-361-3p did not occasion control of the 3′ UTR-containing reporter (Figure 4B). In contrast, transfection of miRs-608-, 155, and -145 reduced mean fluorescence intensity of the 3′ UTR construct compared with GFP alone by approximately 70%, 50%, and 40%, respectively (Figure 4B); this reduction was significant for miR-608.

### Differential expression of putative TTP-targeting miRNAs in vivo

**Figure 5:**
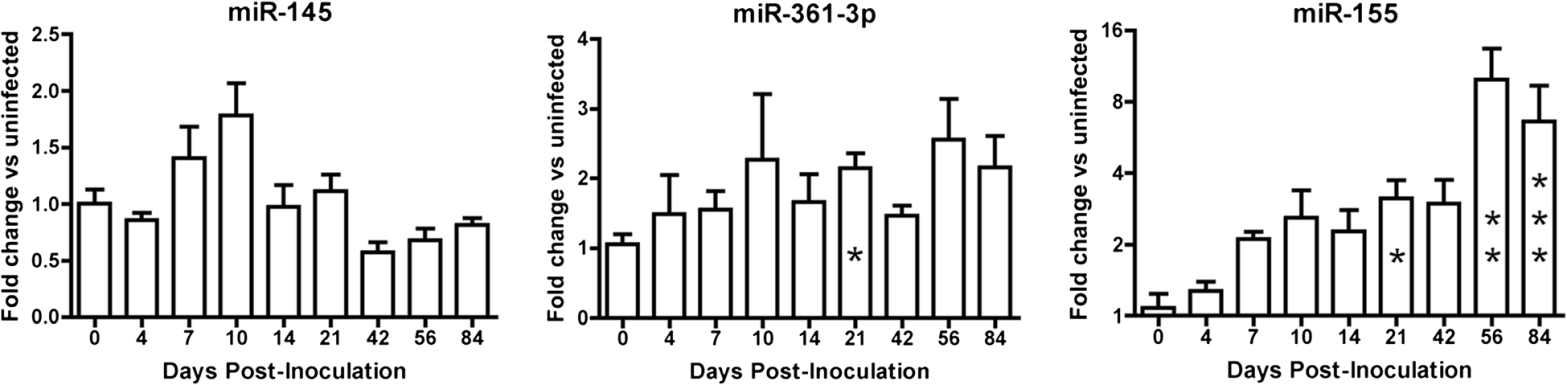
**Longitudinal miRNA expression in SIV-infected brain.** miRs-145, 361-3p, and -155 were measured in thalamus using stem-loop RT-qPCR. Normalization was to U6 snRNA expression, and results are shown in comparison with the average of the control, uninfected (‘0’) samples. Error bars are SEM. Significance of expression differences at each time point versus control was assesssed by Kruskal-Wallis test with Dunn’s multiple comparison test: * (p < 0.05), ** (p < 0.01), *** (p < 0.001).

To evaluate the possible contributions of these miRNAs to TTP regulation, we performed qPCR measurement of miRs-145, -155, -361-3p, and -608 in brain. miR-361-3p and miR-155 were upregulated over the course of infection (Figure 5). The substantial and significant increases in the latter are consistent with the reported roles of miR-155 in inflammation, and miR-155 reached its highest levels in late-stage individuals that had severe CNS disease. miR-608 was detected by PCR and appeared to decline during late-stage disease (data not shown), but its abundance in brain was quite low. Amplification of miR-608 only after PCR cycle 35 in most samples diminished confidence in the accuracy of measurement. In contrast, there was a trend towards miR-145 downregulation during late-stage disease that coincided with increased TTP protein production during this phase (Figure 5). However, although the median abundance values were significantly different during each phase of infection, miR-145 downregulation was not significant according to a Kruskal Wallis test. There was a negative correlation of miR-145 with TTP abundance across time points. This relationship approached but did not reach statistical significance at alpha = 0.05 (R = 0.31, p = 0.055). Nevertheless, these results indicate that miR-145 may play a direct or indirect role in TTP regulation, and that this possibility should be investigated further.

## Discussion

Tristetraprolin transcript was generally downregulated over the course of SIV infection and CNS disease development. Downregulation of TTP mRNA in the acute stage of infection is consistent with the contemporaneous upregulation of numerous cytokines. However, control of cytokine production at the end of acute phase infection [12] appears to involve mechanisms other than TTP upregulation, since TTP levels remained comparatively low during acute and latent infection. On average, during late stage infection, TTP mRNA levels returned to the levels found in uninfected control subjects, but this result was driven largely by large increases of TTP transcript in brain of subjects with the most severe CNS disease.

The mechanism of TTP downregulation likely involves miRNAs. However, our confirmation and extension in primary cells of a previous report that miR-29a exerted regulation through the TTP 3′UTR was not accompanied by observable negative correlations of miR-29a and TTP expression in brain. Indeed, with the exception of increased abundance in several late-stage subjects, particularly those with severe CNS disease, miR-29a expression did not change drastically during the course of infection. We would like to note, however, that we could not perform longitudinal sampling on the same individuals; because of interindividual variation, it remains possible that changes within the individual brain occurred but could be observed only with much larger numbers of subjects.

Since differences in miR-29a levels did not appear to account for TTP regulation during SIV infection, several additional miRNAs were investigated that, out of multiple candidates, exerted regulatory effects through the TTP 3′ UTR. Quantitation of miRs-145, 155, 361-3p, and -608 in brain and comparison of their abundance with that of TTP indicated that miR-145 should be investigated further as a candidate for natural regulation of TTP during SIV infection. The evidence did not support a consistent role for miRs-155, -361-3p, or the very low-abundance miR-608 in TTP regulation. miR-608 did, however, appear to be a potentially effective regulator of TTP in supraphysiologic quantitites.

It may be important to stress that absolute abundance of a miRNA is not the only determinant of its involvement in regulatory processes [41,42]. In addition to factors such as sequestration and differential binding partners, the balance of all targeting miRNAs and all affected target—not simply the level of a single miRNA—may be decisive in determining the magnitude of observed regulation. Numerous miRNAs with predicted MREs in the TTP 3′ UTR are also predicted to target cytokines that are differentially expressed during stages of infection in our model: miR-145 (IL6, TNF-alpha, IFN-beta, and the chemokine CCL2); miR-361-3p (IL10 and MIP1-alpha); miR-29 family members (IFN-gamma, IL12p40); and miR-608 (IL6, TNF-alpha, IL12p35 and -40, IL10, MIP1-alpha and -beta, and CCL2).

We thus posit the following model. During acute infection, TTP transcript abundance is slightly reduced. This likely occurs at the transcriptional level, since ERK activation, which occurs during early viral replication [43], including in our model [44], downregulates TTP [45]; however, a post-transcriptional component such as increased turnover of the TTP mRNA cannot be ruled out. At the same time, miRNA-mediated control of TTP translation is relieved by a combination of mechanisms in the broader context of antiviral innate immune responses. These may include both downregulation of some targeting miRNAs and greatly increased transcription of mRNAs of cytokines and other factors produced during the innate immune responses. These transcripts would act as “sponges” for miRNAs that also target TTP. As a result, TTP protein levels could be maintained or even rise despite lower levels of transcript, and this situation would be exacerbated with increasing transcription. The overall abundance of all mRNA targets of TTP-targeting miRNAs would determine the extent to which miRNA levels could affect TTP production. The explosion of dysregulated cytokine transcripts during progression to CNS disease would thus explain why even greatly increased levels of TTP targeters such as miR-155—or, in some individuals, miR-29a—would have little or no effect on TTP. Carefully integrated systems biology experiments and new approaches for detecting miRNA-target antagonism patterns [46-48] will help to address these issues in future studies.

Apart from the remaining questions regarding mechanisms of TTP regulation, our results show that TTP transcript abundance alone cannot explain changes in cytokine mRNA levels during progression to lentiviral CNS disease, and that increases in TTP are in fact associated with disease development. During late-stage SIV CNS disease in our model, both TTP and its targets, including inflammatory cytokines, are upregulated in brain of individuals with severe encephalitis. It would thus appear that although TTP may influence cytokine levels during early infection, and while the protein likely continues to exert a dampening effect throughout infection, increases in TTP are insufficient to control inflammation during late stage disease. Preliminary results indicate that this may be due to a marked increase in phosphorylated TTP [49] during progression to disease (JL and KWW, unpublished observations). Since phosphorylated forms of the protein are known to have impaired ability to cause mRNA decay [36,50], the prevalent form of the protein during late-stage infection may not effectively regulate transcripts of inflammatory mediators.

The finding of upregulated but partly ineffective TTP in disease is also consistent with reports on TTP in several diseases and models. In an interesting recent study with some parallels to this work, Zhang et al reported that TTP was upregulated in endothelial cells in contact with atherosclerotic lesions [51]. Also, in rheumatoid arthritis upregulated TTP is found in cells involved in pathogenesis [52] and may be a marker of severe disease [53], in part because of production stimulated by the TTP target TNF. Although the complete absence of TTP (knockout mice) is associated with uncontrolled inflammatory responses [20,54-56] and TTP influences cytokine levels in cell culture models, there are conflicting reports on whether TTP and TNF levels are correlated in rheumatoid arthritis patient samples [34,53]. Exposure of mouse brain to hypertonic insult resulted in upregulation of both TTP and TNF [57]. In some cases, then, the normal TTP anti-inflammatory mechanism, although duly activated, cannot completely or on its own stem the rising inflammatory tide of disease- or injury-related dysregulation.

Indeed, TTP can promote apoptosis independently of its effects on mRNA stability, an effect that has prompted the label of “anti-oncogene” [35,58,59]. Thus, the high levels of TTP observed in macaque brain during late stage lentiviral infection may not be protective, but may instead contribute to CNS disease. Increasing the levels of TTP to enable the mRNA-destabilizing activity of this RNA binding protein—for example, through the use of inhibitors of anti-TTP miRNAs [24]—is an attractive therapeutic possibility in cancers [24], atherosclerosis [51], and perhaps other conditions. Models such as ours may play an important role in investigating the possibly deleterious effects of increased TTP in the CNS during lentiviral infection.

## Conclusions

TTP mRNA levels were modulated during SIV infection, but large fold upregulation of TTP was insufficient to control dysregulated CNS cytokine expression during late-stage disease. We also confirmed TTP as a target of miR-29a, but found that higher miR-29a expression in brain was not associated with reduced TTP abundance. The results presented here are consistent with targeting of TTP by additional miRNAs and the existence of a complex interaction network governing TTP, miRNA, and cytokine expression. Additional research is needed to characterize this network more fully, including the contributions of protein levels and post-translational modifications of TTP. Finally, the possibly deleterious effects of increased levels of TTP should be assessed carefully along with any investigation of TTP-altering therapies as a strategy to control cytokine deregulation in retrovirus-associated CNS disease.

## Methods

### Animal studies and ethics statement

No new animal studies were conducted for this project. Archived samples were from previous studies involving dual-inoculation of *Macaca nemestrina* with an immunosuppressive SIV swarm and a neurotropic clone [14]. All animal studies were approved by the Johns Hopkins University Institutional Animal Care and Use Committee and conducted in accordance with the Weatherall Report, the Guide for the Care and Use of Laboratory Animals (NIH Publications No. 80-23, revised 1996), and the USDA Animal Welfare Act.

### Cell culture

HEK-293 T cells were cultured in D10 media (DMEM, 10% fetal bovine serum, 2 mM L-Glutamine, 10 mM HEPES. Cells were passaged upon reaching 80% confluency, approximately every 3 days.

Human macrophages were differentiated from monocytes in peripheral blood mononuclear cells (PBMCs) that were isolated by standard Percoll protocol, as described previously [60]. PBMCs were cultured in macrophage differentiation medium with 20% serum [MDM20: Dulbecco’s modified Eagle’s medium (DMEM, Invitrogen), 20% human serum (GemCell), 2 mM L-Glutamine (Sigma), 10 mM HEPES (Gibco), 10 mM sodium pyruvate (Sigma), 50 ng/ml M-CSF (R&D), and 2 mg/ml Gentamicin (Gibco)] for one week, with a half-volume re-feeding at three days post-plating. After seven days, supernatants were removed and adherent macrophages were washed with PBS. Medium was replaced with MDM10 (MDM with 10% human serum).

### Reporter plasmid cloning

The TTP 3′UTR was amplified from cDNA using primers 5′-GGA CTC AGA TCT CGA GCA AAG TGA C 3′ and 5′ – GAT CCC GGG CCC GCG GTA CCG ATC C-3′ that contained Kpn1 and XhoI restriction sites, respectively. The PCR product was sequenced (GeneWiz), verified against the Ensembl Genome database, and cloned by TOPO cloning kit (Invitrogen). The pEGFP-TTP UTR construct was made by subcloning the TTP3′ UTR into the pEGFP-C1 plasmid (Clontech) using the same restriction enzymes.

### Transfections

HEK-293 T cells were reverse transfected by seeding in antibiotic-free medium onto 96-well plates containing Lipofectamine 2000 (Invitrogen) complexes containing 50 nM synthetic miRNA mimics, miRNA antagonists, or control RNAs (Qiagen) and 150 ng reporter plasmid DNA. Medium was removed, cells were washed with PBS, and fresh medium was added after four hours.

Human monocyte-derived macrophages were transfected with 100 nM synthetic RNAs using Lipofectamine 2000 in medium without antibiotics. Transfection complexes were removed, and cells were washed with PBS and re-fed with fresh MDM10 after 4 hours.

### RNA isolation

RNA was isolated from cells using TRIzol reagent (Invitrogen) per manufacturer’s protocol. Macaque thalamus tissue was homogenized using bead Lysing Matrix D (MP Biomedicals) and TRIzol.

### Flow cytometry

HEK-293 T cells transfected with GFP-containing plasmid DNA, RFP transfection normalization control, and synthetic RNAs were harvested 24 hours post-transfection via trypsinization. Flow cytometry was performed with a BD FACSCalibur instrument. Analysis was performed with Flowjo 9.1 software (Flowjo).

### Quantitative RT-PCR

Reverse transcription quantitative real-time PCR for TTP messenger RNA was performed using macaque brain RNA. Random primers were used to reverse transcribe RNA into cDNA. TTP cDNA was then amplified using the primers 5′-GGC GAC TCC CCA TCT TCA AT-3′ and 5′CAG TGC AAC AGA CGT GGC TC-3′. Amplification was monitored in real time with a HEX-conjugated probe (5′-TCT GAG TGA CAA GTG ACT GCC CGG TCA G-3′). All primers and probes were from IDT. Fold changes in induction were analyzed using the delta-delta-C(t) method with comparison to 18S rRNA as described previously [12]. Cytokine measurements for the same samples were retrieved from our database and were made as described previously [12].

MicroRNA RT-qPCR was performed using the stem-loop RT primer/Taqman microRNA PCR system (Applied Biosystems) [61]. The suggested protocol was followed as previously described [62] with the following alterations. Reverse transcription primer amount was reduced to 1.5 ul per reaction, and reverse transcription reactions were multiplexed. Real-time probe volume was reduced to 0.5 ul per reaction. Fold changes in induction were analyzed using the delta-delta-C(t) method with comparison to the small RNA U6 (as depicted) or, with similar results, to the geometric mean of six relatively invariant small RNAs (data not shown). For both mRNA and miRNA RT-qPCR, amplification reactions were performed on either the iQ5 or the Chromo4 optical system (BioRad).

### Target predictions

A Perl program described previously [13] was used to combine the RNAhybrid [63], TargetScan [64], and MiRanda [65] target prediction algorithms, assigning a score to candidate macaque miRNA/3′ UTR pairs by the number of programs predicting interaction and the number of calls per 3′ UTR.

## Abbreviations

HIV: Human immunodeficiency virus; SIV: Simian immunodeficiency virus; CNS: Central nervous system; AIDS: Aquired immunodeficiency syndrome; HAND: HIV-associated neurocognitive disorder; TTP: Tristetraprolin; 3′ UTR: 3′ untranslated region; ARE: AU-rich element; miRNA: microRNA; MRE: microRNA recognition element; PBMC: Peripheral blood mononuclear cell; GFP: Green fluorescent protein.

## Competing interests

The authors declare that they have no competing interests.

## Authors’ contributions

All authors participated in designing experiments. JL, JMS, and KWW performed the experiments. JL, JEC, and KWW analyzed the data. JEC and KWW obtained funding. JL, JEC, and KWW wrote the manuscript. All authors read and approved the final manuscript.

## Supplementary Material

Additional file 1: Figure S1 Effect of synthetic mir-29a and antagonists on TTP protein production in primary macrophages. Monocyte-derived macrophages at day seven of differentiation were transfected with miR-29a mimic or antagonist, or control mimics or antagonists. Protein was harvested at least 24 hours after transfection and subjected to Western blotting. TTP band intensities were normalized to those of beta-III tubulin. Only a slight reduction of TTP protein was observed with addition of exogenous miR-29a, but a four-fold increase resulted from transfection of miR-29a antagonist. Note that the three middle lanes on the protein blot (not shown in the quantitation graph) represent experiments with miRNA mimics that were unrelated to miR-29a. Click here for file
