# Supplementary figures and images for: Tristetraprolin expression and microRNA-mediated regulation during simian immunodeficiency virus infection of the central nervous system
Source: Mol Brain. 2013 Sep 2;6:40. doi: 10.1186/1756-6606-6-40 (PMC3766027; doi:10.1186/1756-6606-6-40)

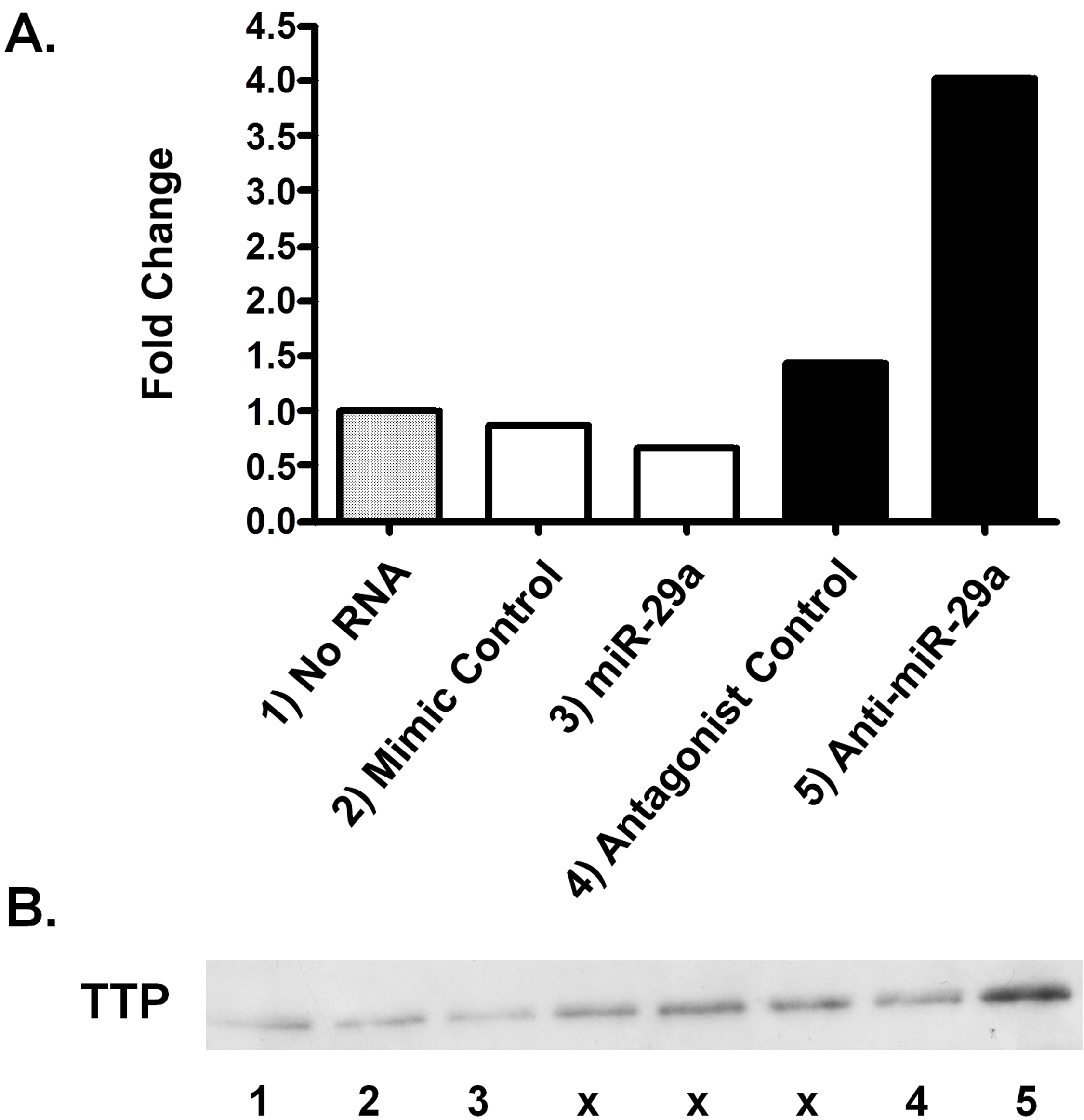

Supplement: Additional file 1: Figure S1 — Effect of synthetic mir-29a and antagonists on TTP protein production in primary macrophages. Monocyte-derived macrophages at day seven of differentiation were transfected with miR-29a mimic or antagonist, or control mimics or antagonists. Protein was harvested at least 24 hours after transfection and subjected to Western blotting. TTP band intensities were normalized to those of beta-III tubulin. Only a slight reduction of TTP protein was observed with addition of exogenous miR-29a, but a four-fold increase resulted from transfection of miR-29a antagonist. Note that the three middle lanes on the protein blot (not shown in the quantitation graph) represent experiments with miRNA mimics that were unrelated to miR-29a. [file 1756-6606-6-40-S1.tiff]
